# Functional Polymorphism of the Mu-Opioid Receptor Gene (OPRM1) Influences Reinforcement Learning in Humans

**DOI:** 10.1371/journal.pone.0024203

**Published:** 2011-09-02

**Authors:** Mary R. Lee, Courtney L. Gallen, Xiaochu Zhang, Colin A. Hodgkinson, David Goldman, Elliot A. Stein, Christina S. Barr

**Affiliations:** 1 National Institute on Drug Abuse, Intramural Research Program, Baltimore, Maryland, United States of America; 2 National Institute on Alcohol Abuse and Alcoholism, Intramural Research Program, Rockville, Maryland, United States of America; University of Granada, Spain

## Abstract

Previous reports on the functional effects (i.e., gain or loss of function), and phenotypic outcomes (e.g., changes in addiction vulnerability and stress response) of a commonly occurring functional single nucleotide polymorphism (SNP) of the mu-opioid receptor (OPRM1 A118G) have been inconsistent. Here we examine the effect of this polymorphism on implicit reward learning. We used a probabilistic signal detection task to determine whether this polymorphism impacts response bias to monetary reward in 63 healthy adult subjects: 51 AA homozygotes and 12 G allele carriers. OPRM1 AA homozygotes exhibited typical responding to the rewarded response—that is, their bias to the rewarded stimulus increased over time. However, OPRM1 G allele carriers exhibited a decline in response to the rewarded stimulus compared to the AA homozygotes. These results extend previous reports on the heritability of performance on this task by implicating a specific polymorphism. Through comparison with other studies using this task, we suggest a possible mechanism by which the OPRM1 polymorphism may confer reduced response to natural reward through a dopamine-mediated decrease during positive reinforcement learning.

## Introduction

Mu-opioid receptors are located throughout extended brain circuits involved in positive reinforcement and are critical in processing reward, analgesia, and stress responses, reviewed in [1]. Given the important role of this receptor in motivational states, natural reward, and the development of addiction [2], there has been interest in identifying variations in the mu-opioid receptor gene that predict individual differences in reward processes and potentially addiction vulnerability [3].

A commonly occurring non-synonymous SNP (rs1799971; OPRM1 A118G) results in an amino-acid substitution (Asn40Asp or N40D) in the N-terminal region of the mu-opioid receptor. The polymorphism was originally shown to confer a 3-fold increase in affinity for the endogenous ligand, ß-Endorphin [4], although more recent studies have shown the effects of the G allele on affinity for ß-Endorphin to be cell-line dependent, and further, that variant, G-allele, receptors exhibit significantly lower cell-surface receptor binding site availability compared with the prototype 118A receptor [4]–[6]. Further, using a knock-in mouse model of the human A118G SNP, Mague et al. [7] found decreased mRNA expression and receptor protein levels in mice heterozygous for the G allele. Similarly, a human post-mortem study [8] also found decreased mRNA in G118 allele carriers. Taken together, the precise functional consequence of this variant in humans remains unclear.

Although much of the work on the effects of the OPRM1 polymorphism has been in the addiction field, particularly alcohol–induced euphoria, dependence, and treatment response [2], more recent studies suggest that functional OPRM1 genotypes may have been under evolutionary selection [9] and that, in addition to drug-induced reward, they may also moderate responses to natural rewards [10], [11]. For example, a similar functional polymorphism of the OPRM1 gene in nonhuman primates [12]–[14] increases mother-infant attachment after separation [10], potentially reflecting increased genotype-mediated reward sensitivity. Studies in humans have also extended this polymorphism's potential role in processing physical and emotional pain. Specifically, carriers of the G allele have been shown to be more sensitive to the pain of social rejection [11] and to the experience of physical pain [15]. Whereas many other studies have examined the effect of genetic variations related to dopaminergic transmission (e.g., COMT and DAT) or of dopamine receptors (e.g., DRD2 and DRD4) [16]–[20] on reward processing, studies of OPRM1 A118G suggest that, perhaps through upstream modulation of dopamine, variations in the opioid system may also play a role in moderating responses to natural reward and punishment [2]. To date, however, no studies have directly examined behavioral measures of sensitivity to natural reward in humans as a function of OPRM1 genotype. Assessing the effects of OPRM1 genotype on probabilistic reward learning may yield an intermediate phenotype from which inferences can be drawn about the role of this polymorphism in human reward processing. Accordingly, we used a signal detection task [21] to assess the effects of OPRM1 A118G genotype on reward sensitivity.

## Methods

### Participants

Sixty-three participants were chosen from a nonclinical, diverse group of healthy subjects participating in a larger study involving genotyping and brain imaging. Participants gave written informed consent approved by the National Institute on Drug Abuse-IRP Institutional Review Board. Subjects had no current Axis I diagnoses as measured by the computerized Structured Clinical Interview for *DSM-IV*; substance dependence was confirmed by clinical interview using *DSM-IV* criteria. All participants tested negative for illicit drugs and alcohol before completing the probabilistic reward task. See Table 1 for detailed subject demographics.

**Table 1:** Comparison of demographic variables between genotype groups.

	A Allele Homozygotes	G Allele Carriers
*N*	51	12
Age (mean ± SD)	31.82±9.05	33.67±9.59
Gender (F/M)	29/22	6/6
WASI Vocabulary (mean ± SD)	55.51±7.67	59.5±9.44
Drug use (user/nonuser)	19/32	3/9
BDI (mean ± SD)^1^	2.76±4.58	4.36±5.50
African ethnic factor score (mean ± SD)^2^	0.55±0.40	0.06±0.18
European ethnic factor score (mean ± SD)^3^	0.25±0.36	0.67±0.40
American ethnic factor score (mean ± SD)	0.01±0.01	0.01±0.02
Asian ethnic factor score (mean ± SD)	0.09±0.18	0.07±0.17
Far East Asian (mean ± SD)	0.04±0.19	0.09±0.28
Middle Eastern (mean ± SD)	0.05±0.05	0.09±0.14
Oceanic (mean ± SD)	0.00±0.01	0.02±0.03

There were no genotype group differences between groups in all demographic variables except African and European ethnic factor scores (^2^P<0.001; ^3^P = 0.001). Also note that Ns for BDI scores^1^ were 49 and 11 for A allele homozygotes and G allele carriers, respectively.

### Procedures

#### Response Bias Task

A line discrimination task was modeled after that reported by Pizzagalli et al. [25]. In this signal detection task, participants tend to become biased to respond to a more frequently rewarded stimulus over time [25]. The task consisted of 300 trials divided into 3 blocks of 100 trials each, with blocks separated by a 1–2 minute break. Subjects were instructed to win as much money as possible. At the start of each trial, participants viewed an asterisk in the middle of the screen that served as a fixation point. A mouth-less cartoon face was then presented in the center of the screen for 500 ms and, after a delay, either a short or a long (9 vs. 11 mm) mouth was presented on the screen for 100 ms. The face (without the mouth) then remained on the screen until a response was made by the participant, who was asked to identify which type of mouth was presented (long or short) by pressing one of two keys on a response-box.

For each block, the long and short mouths were presented equally in randomized sequences. For each block, 40 correct trials were followed by reward feedback (i.e., “Correct!! You won 25 cents.”), which was presented for 1750 msec immediately after the correct response. For trials without feedback, participants saw a blank screen for 1750 msec before the next trial. Participants were instructed that not all correct responses would receive feedback. An asymmetrical reinforcer ratio was used for the task feedback in which a certain mouth (e.g., long or short) was given more frequent positive feedback on correct responses than the other. For half of the participants, correct identification of the short mouth was associated with three times more positive feedback (i.e., 30 of 40) than the correct identification of the long mouth (i.e., 10 of 40). For the other half of the participants, the contingencies were reversed.

#### Genetic Analysis

Participants were recruited to participate in this study regardless of genotype and genotyping was completed after enrollment in the study. DNA was extracted from blood using standard protocols. The OPRM1 rs1799971 missense polymorphism, Asn40Asp (A118G), was genotyped using the Illumina GoldenGate platform as previously described [22]. The genotype distribution was as follows: 51 AA homozygotes, 11 AG heterozygotes, 1 GG homozygote. Because of the limited sample size, G allele carriers (AG and GG) were grouped together. This approach is in keeping with precedent in the field [11]. As allele frequencies for A118G differs among human populations, ancestry was determined. A total of 186 ancestry-informative markers (AIMs) [22] were genotyped in our sample and in the HGDP-CEPH Human Genome Diversity Cell Line Panel (1051 individuals from 51 worldwide populations) (http://www.cephb.fr/HGDP-CEPH-Panel). PHASE *Structure 2.2* (http://pritch.bsd.uchicago.edu/software.html) was run simultaneously using the AIMs data from our sample and the 51 CEPH populations to identify population substructure and compute individual ethnic factor scores.

#### Analysis of Behavioral Data

Outcome measures included discriminability and response bias. Discriminability, calculated by log D = ½ log [(Rich_correct_ * Lean_correct_)/(Rich_incorrect_* Lean_incorrect_)], where “Rich_correct_” represents the number of correct responses after presentation of the rich, or more frequently rewarded, stimulus etc. [25], was defined as the ability to discriminate the difference between the two stimuli, a measure of attention and task difficulty. Response bias, calculated by the following formula: log RB = ½ log [(Rich_correct_ * Lean_inorrect_)/(Rich_incorrect_* Lean_correct_)] [25],was defined as a preference for the more frequently rewarded stimulus. High response bias scores corresponded to more correct responses for the more frequently rewarded stimulus (“rich” stimulus) and/or more incorrect responses for the less frequently rewarded stimulus (“lean” stimulus). These measures were assessed in 100-trial increments, forming three blocks from which changes in responding over time could be assessed.

In general, participants tend to ‘drift’ to the rewarded response across time (as assessed by increased in response bias scores in later blocks compared to earlier blocks). As seen in the calculation^2^, participants can increase response bias over time by increasing correct identification of the rewarded (rich) stimulus and/or increasing misclassification of the non-rewarded (lean stimulus) as the rich stimulus. Secondary analyses examined genotype group differences in hit rate responses (percent correct) for lean and rich stimuli across the three task blocks.

## Results

### Subject Characteristics

OPRM1 genotype groups did not significantly differ in age, gender, IQ as measured by the vocabulary subscale of the Wechsler Abbreviated Scale of Intelligence (WASI) [23], depressive symptoms as measured by the Beck Depression Inventory (BDI) [24], or drug use (Table 1). Participants were categorized as ‘users’ if they were daily smokers, dependent on cocaine, or used marijuana (daily use or dependence). There were 4 cocaine dependent subjects, 16 daily smokers, and 2 marijuana users (4 cocaine users, 2 marijuana users and 13 smokers in the AA homozygote group). On the day of the study session, smokers used nicotine in their usual pattern, cocaine users were 4 days abstinent (range 4–20), and marijuana users were 9 and 30 days abstinent. African and European ethnic factor scores were, however, significantly different between genotype groups (Table 1). Thus, African and European ethnic factor scores were used as covariates in all subsequent analyses comparing genotype groups.

### Discriminability

**Figure 1:**
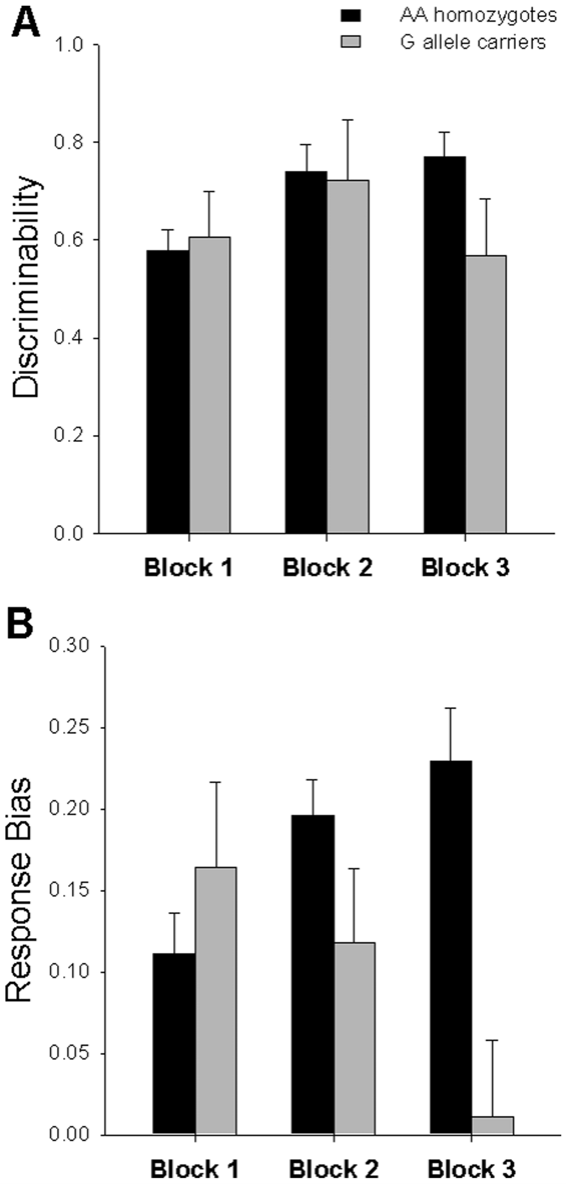
Mean discriminability and response bias across blocks for AA homozygotes and G allele carriers. AA homozygotes (black bars) did not differ from G allele carriers (gray bars) in measures of discriminability (A). There was, however, a genotype group×block interaction for response bias (B), in which AA homozygotes had increased response bias over time, while G allele carriers had decreased response bias over time. Error bars represent standard errors.

A repeated measures ANCOVA with factors of block (within-subjects) and genotype group (between-subjects) showed a significant main effect of block (*F*(2,118) = 5.63, *p* = 0.005). Post-hoc analyses revealed that this effect was due to greater discriminability in block 2 (0.73±0.52) than block 1 (0.59±0.38; *p* = 0.021). There was neither a main effect of genotype group (*F*(1,59) = 0.34, *p* = 0.55) nor block×genotype group interaction (*F*(2,118) = 2.27, *p* = 0.12) (Figure 1A).

### Response Bias

The ANCOVA of block and genotype group showed no main effect of block (*F*(2,118) = 0.48, *p* = 0.62). There was, however, a trend toward a main effect of genotype group (*F*(1,59) = 3.73, *p* = 0.06), with greater response bias in AA homozygotes compared to G allele carriers. There was also a significant block×genotype group interaction (*F*(2,118) = 5.30, *p* = 0.006) (Figure 1B). Specifically, in AA homozygotes, response bias increased from block 1 to block 2 (*p* = 0.003) with no further increase between blocks 2 and 3 (*p* = 0.28). However, in G allele carriers, response bias did not significantly change from block 1 to block 2 (*p* = 0.35), and decreased in block 3 relative to block 1 (*p* = 0.01) and block 2 (*p* = 0.01). An analysis of response bias separate by block revealed that this interaction was driven by genotype group differences in response bias in block 3 only (*F*(1,59) = 8.533, *p* = 0.005), while there were no genotype group differences in response bias in block 1 (*F*(1,59) = 0.07, *p* = 0.79) or block 2 (*F*(1,59) = 1.88, *p* = 0.18).

### Consideration of Potential Confounding Factors

#### Ethnic Factor Scores

Correlations between all subjects' response bias scores (separately by block) and European and African ethnic factor scores revealed no significant associations between these variables (*p* range 0.1 to 0.98). Separate correlations by genotype group revealed no significant correlations with AA homozygotes, and one significant negative correlation between European ethnic factor score and response bias in block 1 for G allele carriers (*r*(11) = −0.59, *p* = 0.04). Despite this significant association between ethnic factor scores and response bias, it is unlikely that this correlation influenced our results, as genotype group differences in response bias were seen in block 3 only and this relationship was observed in block 1.

#### Participant User Status

Although there was not a significant difference in the distribution of users and nonusers between the genotype groups (Fisher's exact test, p = 0.52), it is possible that we did not have enough power to detect such a difference (i.e., with only 3 users in G allele carrier group). Thus, there may be a qualitative difference between genotype groups in terms of user status that is driving the response bias behavior. To ensure this potential confound was not affecting the results, all ANCOVAs (i.e., discriminability and response bias) were repeated, controlling for user status both as two groups (user/non-user) and three groups (user/smoker/non-user). Notably, including these covariates did not significantly change any results.

Further, drug groups (user/smoker/non-user) were assessed on their response bias behavior using repeated measures ANOVA across blocks. There was no interaction between drug group and block (*F*(4,120) = 0.84, *p* = 0.50); however, there was a main effect of drug group (*F*(2,60) = 3.46, *p* = 0.04), in which smokers showed a lower response bias compared to controls (*p* = 0.016) and tended to also show lower response bias compared to users (*p* = 0.064). However, a follow up comparison of drug groups separately by block revealed that this effect was due to drug group differences in block 1 only (*F*(2,60) = 6.05, *p* = 0.004), while there were no drug group effects in block 2 (*F*(2,60) = 2.26, *p* = 0.11) or block 3 (*F*(2,60) = 0.36, *p* = 0.70). Thus, it is unlikely that drug group differences in response bias influenced our results, as differences due to drug group were only observed in block 1 and our main finding of genotype group differences were driven by response bias scores in block 3.

#### Genotype Group Matching

In an effort to further disentangle the effect of OPRM1 genotype from the effects of drug use and ethnicity on response bias, analyses were repeated comparing all G allele carriers (n = 12) to a subgroup of individually matched AA homozygotes (n = 12). Genotype groups did not significantly differ in age (*p* = 0.213), WASI vocabulary score (*p* = 0.819), BDI (*p* = 0.419), gender (*p* = 0.206), or any ethnic factor score (*p* range 0.313 to 0.990, median = 0.625). Further, genotype groups also did not differ in drug use (3 cigarette smokers and 9 nonusers in each group, *p* = 1.0).

A repeated measures ANOVA of discriminability scores showed a trend toward a significant main effect of block (*F*(2,44) = 2.914, *p* = 0.065), with discriminability tending to be lower in block 1 (0.631±0.116) than blocks 2 (0.795±0.367, *p* = 0.057) or 3 (0.705±0.236, *p* = 0.082). There were no significant main effects of genotype group (*F*(1,22) = 0.036, *p* = 0.852) or genotype group×block interactions (*F*(2,44) = 0.656, *p* = 0.524). Note that these results are similar to those described above.

Importantly, a repeated measures ANOVA of response bias scores showed a significant genotype group×block interaction (*F*(2,44) = 6.989, *p* = 0.002), replicating the finding found in the larger sample of participants. Specifically, G allele carriers showed a significant decrease in response bias in block 3 (0.011±0.170) compared to blocks 1 (0.164±0.214, *p* = 0.009) and 2 (0.118±0.204, *p* = 0.012), while AA homozygotes showed no significant changes in response bias across blocks (*p* range 0.137 to 0.382). Further, genotype group comparisons in each block also confirmed the original findings, with G allele carriers showing significantly reduced response bias compared to AA homozygotes only in block 3 of the task (0.011±0.184 vs. 0.259±0.184; *F*(1,22) = 11.110, *p* = 0.003), while there were no genotype group differences in blocks 1 (*F*(1,22) = 0.02, *p* = 0.888) or 2 (*F*(1,22) = 1.406, *p* = 0.248). Notably, these results highlight that the main finding of a reduction in response bias over time in OPRM1 G allele carriers is also evident in a smaller sample that was matched for ethnicity and cigarette smoking and excluded cocaine and marijuana users. Finally, there was also a trend toward a significant main effect of genotype group (*F*(1,22) = 3.065, *p* = 0.094), with AA homozygotes tending to have greater response bias than G allele carriers (0.217±0.166 vs. 0.098±0.166). There was no significant main effect of block (*F*(2,44) = 0.748, *p* = 0.479)

### Hit Rates

ANCOVAs of hit rates (percent of correctly identified stimuli) were completed in a similar fashion to those of discriminability and response bias, although separate ANCOVAs were carried out for lean and rich hit rates across blocks.

#### Rich Hit Rate

**Figure 2:**
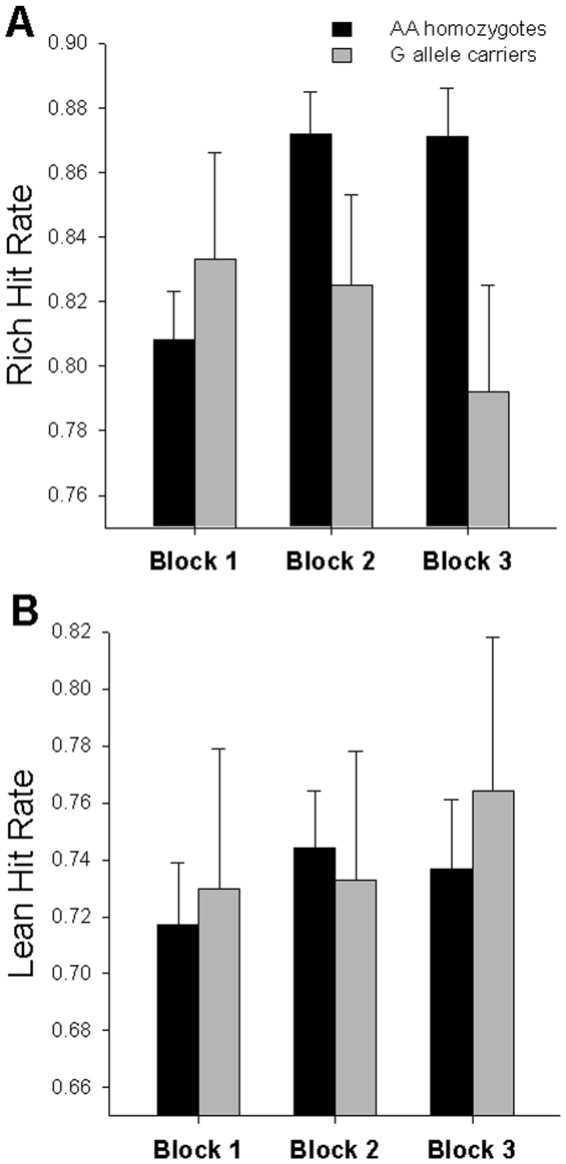
Mean rich and lean hit rates across blocks for AA homozygotes and G allele carriers. There was a genotype group×block interaction for rich hit rates (A). AA homozygotes (black bars) had increased rich hit rates over time while G allele carriers (gray bars) had decreased rich hit rates over time. There were no effects of genotype group or interactions for lean hit rates (B). Error bars represent standard errors.

Rich hit rates were calculated with the formula: Rich_correct_/(Rich_correct_+Rich_incorrect_). The ANCOVA of block and genotype group for rich hit rates showed no main effect of block (*F*(2,118) = 0.09, *p* = 0.92) or genotype group (*F*(1,59) = 1.30, *p* = 0.26). There was, however, a block×genotype group interaction (*F*(2,118) = 5.01, *p* = 0.008) (Figure 2A), similar to that reported for overall response bias scores. Specifically, in AA homozygotes, rich hit rates increased from block 1 to block 2 (*p*<0.001) with no further increase between blocks 2 and 3 (*p* = 0.84). However, in G allele carriers, rich hit rates did not change from block 1 to block 2 (*p* = 0.33), and also decreased in block 3 relative to block 1 (*p* = 0.04) and block 2 (*p* = 0.045). An analysis of rich hit rates separately by block revealed that this interaction was driven by genotype group differences in rich hit rates in block 3 only (*F*(1,59) = 4.55, *p* = 0.04), while there were no genotype group differences in rich hit rates in block 1 (*F*(1,59) = 0.47, *p* = 0.50) or block 2 (*F*(1,59) = 2.19, *p* = 0.15).

#### Lean Hit Rate

Lean hit rates were calculated with the formula: Lean_correct_/(Lean_correct_+Lean_incorrect_). The ANCOVA of block and genotype group for lean hit rates showed no main effect of block (*F*(2,118) = 1.19, *p* = 0.31) or genotype group (*F*(1,59) = 0.04, *p* = 0.84) and no interaction between block and genotype group (*F*(2,118) = 0.32, *p* = 0.73) (Figure 2B).

## Discussion

We used a probabilistic reward task to determine the effects of OPRM1 genotype on response bias (i.e., tendency to drift to a rewarded response over time as a result of positive reinforcement learning). We found that, while AA homozygotes displayed typical reward sensitivity by drifting to the rewarded response, carriers of the OPRM1 G allele exhibited aberrant reward responding by showing a significant decline in response bias over time. These findings were not due to group differences in task performance, such as the ability to discriminate between the two stimuli in the task. We further examined the way in which subjects developed response bias on this task: correct classification of the rich (more frequently rewarded) stimulus and/or misclassification of the lean (less frequently rewarded) stimulus. We found that the deficit in response bias in G allele carriers was solely due to decreased accuracy in identifying the rich stimulus during the third block of the task rather than increased accuracy identifying the lean stimulus. In other words, in G allele carriers, there was a reduced response specifically to the more rewarded stimulus on the final block of the task rather than a global decrease in performance. In addition to providing the first demonstration for the effects of this polymorphism on reward learning, our results may suggest a putative mechanism by which OPRM1 A118G confers its functional effects.

Previous work using this probabilistic reward task in clinical as well as nonclinical populations has shown that reduced reward responsiveness is associated with and predictive of anhedonia in a nonclinical population [25], diagnosis of depression in a clinical population [26], elevated perceived stress [27], and pharmacologically induced reduction of dopaminergic transmission [28]. In the latter study, Pizzagalli et al. [28] found that low doses of a dopamine agonist reduced rewarded responding in this task and that this deficit was due to reduced accuracy for the rewarded stimulus in later blocks, a similar pattern to what is reported here for G allele carriers. The authors have further shown (with computational modeling) that low doses of a dopamine agonist reduce phasic dopamine bursts through presynaptic inhibition [29], which may impair reinforcement learning by reducing the phasic “Go” signal during positive feedback [28], [29]. In contrast, depressed subjects [26] demonstrate a different pattern of performance on this task, in which the reduction in response bias is a consequence of a global response reduction to all stimuli (i.e., both a reduced response to the rich stimulus and failure to misclassify the lean stimulus).

Our study demonstrates that OPRM1 G allele carriers show aberrant reward responding, similar to that observed with reduced phasic dopamine signaling, rather than that seen in individuals with clinical depression. This suggests that the OPRM1 polymorphism may confer similar functional effects to a low-dose dopamine agonist by reducing phasic dopamine signaling during positive feedback. There is a well characterized modulation of dopaminergic signaling by the endogenous opioid system [30], [31]. Specifically, activation of mu-opioid receptors on GABA interneurons in the ventral tegmental area (VTA) leads to decreased firing of GABA neurons and, thus, decreased GABA release. In turn, activation of GABA receptors is decreased, which ultimately results in increased dopamine signaling in the nucleus accumbens (NAc). By extension, decreased opioid signaling in the VTA results in lower levels of dopamine cell firing and reduced dopamine release in the NAc. Further, mu-opioid receptor gene knockout mice exhibit increased GABAergic input [32] and thus decreased firing frequency of midbrain dopamine neurons [33]. With respect to natural reward, opioid peptides and dopamine interact within the VTA-NAc pathway to regulate various aspects of natural reward, such as feeding [1] and sexual behavior [34]. Thus, if the diminished reinforcement learning in G allele carriers reported here is a result of reduced phasic dopamine signaling, this may be directly caused by decreased mu-opioid receptor activation in G allele carriers and increased GABA neuron firing and release in the VTA.

The possibility of decreased opioid receptor activation and signaling in individuals with the G allele variant may also be evident in human studies examining the effects of the OPRM1 A118G polymorphism on responses to negatively valenced stimuli, such as physical pain [15], emotional rejection [11] and stress [35]. Collectively, these studies show augmented behavioral responses to aversive stimuli in G allele carriers. In pain states, there is an associated release of endogenous opioids in midbrain and cortical areas, resulting in activation of mu-opioid receptors and reduction in the subjective experience of pain [36]. Increased sensitivity to physical and emotional pain in G allele carriers, therefore, may be related to reduced release of endogenous opioids and activation of mu-opioid receptors during aversive states.

This study has several limitations. First, the sample size for a genotyping experiment is small. However, the task probes an intermediate phenotype and, as such, behavior on this task may be more closely linked to expression or functional effects of genes than is observation or behavioral reports. Similarly small samples have identified other genotype effects using refined intermediate phenotypes [37], [38] and, further, the effect size [39] of the genotype group differences in response bias in block 3 was large (Cohen's *d* = 1.07). Second, this is a narrow laboratory-based task, and it is unclear how response bias extends to behavior in the “real world.” Despite these limitations, this study extends previous work by identifying a specific genetic variant that influences performance on this task. Further, the results here add to the literature on the effects of variations in dopaminergic genes on reward processing, by suggesting a mechanism through which the opioid system may also play an important role in moderating similar processes, through the OPRM1 A118G substitution. Further research into the effects of this common nonsynonymous variant on learning in the context of negative feedback may shed light on the complex and, in some cases, contradictory findings within the accumulated behavioral data associated with the OPRM1 polymorphism.
